# Mitigating the light pollution problem via spectral adjustment: color-biased phototaxis in male glow-worms

**DOI:** 10.1007/s00442-025-05768-3

**Published:** 2025-07-12

**Authors:** Linnea Kivelä, Christina Elgert, Topi K. Lehtonen, Ulrika Candolin

**Affiliations:** 1 Organismal and Evolutionary Biology, University of Helsinki, PO Box 65, 00014 Helsinki, Finland; 2 Tvärminne Zoological Station, University of Helsinki, J.A. Palménin Tie 260, 10900 Hanko, Finland

**Keywords:** Artificial light at night, Lampyridae, Light attraction, Orientation, Spectral tuning

## Abstract

Light pollution is a widespread threat to dark-adapted species. Attraction to artificial light can have severe, even fatal, consequences with population level repercussions, especially for nocturnal insects. Since most insects are particularly drawn to short-wavelength light, the recent switch to LEDs with a greater emission in the blue range has exacerbated the problem. One potential mitigation measure is to adjust light spectra toward longer wavelengths. However, the effectiveness of this measure may vary among species that differ in their spectral sensitivities. In glow-worms (*Lampyris noctiluca*), nocturnal beetles with presumably declining abundance, especially blue and white artificial light hamper males’ search of yellowish-green glowing females. Here, we assessed whether easing the search by switching to longer wavelengths impacts males’ phototactic behavior. We recorded the movements of males in an arena illuminated at one end by either white, yellow, red, or no artificial light. Males displayed positive phototaxis toward yellow and red light, and negative phototaxis toward white light, with the latter also being associated with reduced activity. Therefore, males' attraction to longer wavelengths that resemble the female glow could be an evolutionary trap in human-modified environments with artificial lights of that color range. These results show that different wavelengths can influence disparate behaviors even within a single species and adjustment of outdoor lighting systems, by filtering out the blue part of the spectrum, has limited capacity to solve the light pollution problem.

**Supplementary Information:**

The online version contains supplementary material available at 10.1007/s00442-025-05768-3.

## Introduction

Artificial light at night (ALAN) is a globally widespread threat to nocturnal species due to its influence on behavior and physiology (Falchi et al. 2016; Kyba et al. 2017; Gaston et al. 2021). Light pollution impacts a wide range of both terrestrial and aquatic species, with most documentation on birds, bats and insects (Rodrigo-Comino et al. 2023). Impacts on insects are of particular interest, as light pollution has probably contributed to their global decline (Grubisic et al. 2018; Owens et al. 2020). Artificial light can compromise spatial orientation, timing of activities, ability to discriminate colors, and the visibility of cues and signals, such as those based on bioluminescence and used in social interactions and mate attraction (reviewed in Owens & Lewis 2018). Attraction to artificial light has received particularly extensive attention, because it can have severe fitness consequences (Frank 2006; van Langevelde 2011; Macgregor et al. 2015; Perkin et al. 2014; Kühne et al. 2021) . For instance, approximately one-third of insects drawn to streetlights suffer direct mortality, and further costs may arise from injury, increased predation risk and entrapment (Eisenbeis 2006; Frank 2006; Haynes & Robertson 2021). 

Light of short wavelength (blue and UV) is particularly attractive to a wide range of taxa, such as many insects (Deichmann et al. 2021), anuran amphibians (Hailman & Jaeger 1974; Buchanan 2005) and sea turtle hatchlings (Witherington & Bjorndal 1991). In addition, short-wavelength light disrupts circadian rhythms in humans and many other vertebrates (Grubisic et al. 2019) . Thus, yellow and red lights have been proposed as more environmentally friendly alternatives for outdoor use (Longcore & Rich 2016; Spoelstra et al. 2017; Longcore et al. 2018). On one hand, the recent switch to broad spectrum light-emitting diodes, LEDs, with higher emission in the blue part of the spectrum, has exacerbated the light pollution problem (Sánchez de Miquel et al. 2021). On the other, the large-scale use of LEDs has overcome technological limitations of the past regarding adjustment of spectral properties of artificial light, by allowing spectral tuning via filtering out specific wavelengths (Pimputkar et al. 2009; van Grunsven et al. 2019; Pagden et al. 2020; Martín et al. 2021). 

Given the increased availability of technical solutions, the color of light has received recent interest in pest management (Shimoda & Honda 2013; Park & Lee 2017; Kim et al. 2019) and in the mitigation of ecological impacts of light pollution (van Langevelde 2011; Donners et al. 2018; Owens et al. 2022; Spoelstra et al. 2023), while its ecological effects have nevertheless remained poorly understood. Notably, spectral sensitivities may vary between species (Wakunawa et al. 2014; Pacheco et al. 2016; Park & Lee 2017; Kühne et al. 2021; Pan et al. 2021), as well as life stages (Carleton et al. 2008; Frank et al. 2009; Futahashi et al. 2015; Kühne et al. 2021) and the sexes (Arikawa et al. 2005; Bloch 2015; McCulloch et al. 2017) within a species. For example, the visual system of most insects is based on three types of photoreceptors: UV-, blue- and green-sensitive, whereas a small number of species also have red photorepectors, with spectral sensitivity being further modified by, e.g., visual, filtering and screening pigments, chromophores, and their spatial arrangements (Briscoe & Chittka 2001). Moreover, different wavelengths may influence disparate behaviors of a single organism (Leech et al. 2005; Paskin et al. 2014). Therefore, we need more research on spectral adjustment of artificial lights as a solution to the light pollution problem.

*Lampyris noctiluca,* the common glow-worm of Europe, is a nocturnal beetle in the firefly family Lampyridae that is sensitive to the color of artificial light (Kivelä et al. 2023). At night, flightless females emit a yellowish-green bioluminescent light (peak ~ 550 nm, range 500–700 nm; De Cock 2004; Borshagovski et al. 2020) to lure flying males. Artificial light inhibits female glowing (Elgert et al. 2020) and decreases the ability of males to detect females (e.g., Bird & Parker 2014; Elgert et al. 2020; Stewart et al. 2020; Van den Broeck et al. 2021a, b). In this respect, light of shorter wavelength (blue and white) has a stronger negative effect than light of longer wavelength (yellow and red) (Kivelä et al. 2023), which suggests that switching to longer wavelengths in outdoor lighting could reduce the negative effects of ALAN. However, anecdotal evidence indicates that glow-worm males are attracted to yellow and red light (Schwalb 1961). Hence, different behaviors may be sensitive to different colors of light: while female glowing and male mate detection are compromised by shorter wavelengths, male orientation might also vary with light color, potentially resulting in entrapment by sources of longer wavelength light. If so, the adjustment of the spectrum of artificial light to longer wavelengths may not benefit the species overall.

To investigate whether different behaviors can be sensitive to disparate spectra, we performed a laboratory experiment that investigated the phototaxis of male glow-worms in relation to artificial light of different colors. We expected males to be more attracted (i.e., to exhibit positive phototaxis) toward light dominated by longer wavelengths (yellow and red) than light with a major short-wavelength component (white), the type currently used in most outdoor LED lighting systems.

### Materials and methods

We collected male glow-worms from the proximity of Tvärminne Zoological Station in Southern Finland (N 59°51′, E 23°14′) on 17 nights between 11-Jun-2021 and 2-Jul-2021, using live capturing traps with dummy female lures (for construction, see Hopkins et al. 2015; Lehtonen & Kaitala 2020; Kivelä et al. 2023).

After capture, we kept the males indoors in transparent jars (exposed to natural light through a large window or an open door) until the following night's experiment. We marked each male with a small dot of acrylic color on its pronotum (first exoskeletal shield) and then transferred it to a single-individual plastic jar. When the traps had captured a larger number of males than was required for the night’s experiment, we released the excess males. We also released males used in the experiment the following day. The color markings allowed us to identify any recaptured males and exclude them from being reused in the experiment.

**Fig. 1:**
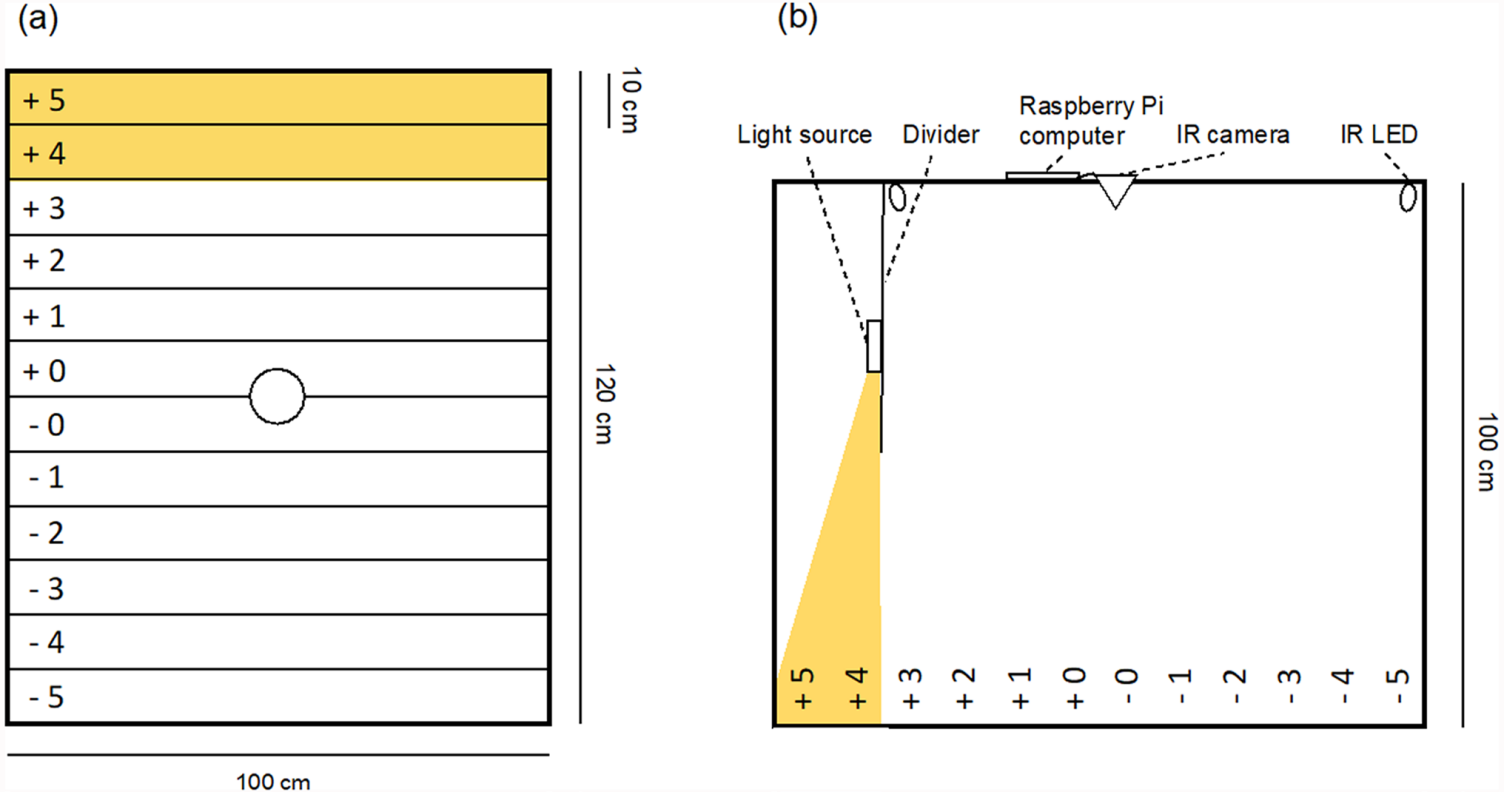
Schematic illustration of the experimental arena from **a** above and **b** side

We investigated male orientation in relation to artificial light in an experimental arena of 120 × 100 × 100 cm (length × width × height) made of hardboard. The arena had doors at both ends through which males could be put in and taken out. The floor was covered with white paper and longitudinally sectioned into twelve 10 cm wide “zones” (Fig. 1a). The zones were labeled from + 5 to -5, with the “zero” zones (+ 0 and -0) in the center of the arena. The furthest 20 cm of the arena (zones + 4 and + 5) were illuminated by artificial light, using a Fenix UC01 mini LED flashlight (at its lowest output) that was covered with EUROLITE color-foil to alter the light spectrum (see Table 1, Online Resource 1). A cardboard divider (along the width of the arena, height: 40 cm) was attached to the ceiling, at 20 cm distance from one end of the arena (Fig. 1b). The light source was attached onto the divider so that focal males were not able to see it outside zones + 4 and + 5 (Fig. 1b). Light was directed toward the bottom of the arena, and the divider also limited the cone of light and created a steep alteration in light intensity between zones + 3 and + 4 (Online Resource 2).

**Table 1:** Light intensities under the different light conditions (treatments) and at different distances (zones) from the central dish

		Zone						
Color	Peak	+ 4/ + 5	+ 2	0	-2	-4/-5		
White	450 nm	3.52E + 12	4.07E + 11	2.29E + 11	1.14E + 11	6.10E + 10	Photons cm^−2^ s^−1^	Intensity
Yellow	587 nm	3.74E + 12	5.17E + 11	2.83E + 11	1.48E + 11	1.04E + 11		
Red	617 nm	3.83E + 12	6.68E + 11	3.81E + 11	2.20E + 11	1.67E + 11		
Control	–	< 1E + 9	< 1E + 9	< 1E + 9	< 1E + 9	< 1E + 9		
White	450 nm	3.47	3.79E-01	2.02E-01	9.49E-02	5.38E-02	Lux	
Yellow	587 nm	4.72	5.45E-01	2.93E-01	1.43E-01	9.02E-02		
Red	617 nm	2.51	3.60E-01	2.01E-01	1.08E-01	7.08E-02		
Control	–	< 0.001	< 0.001	< 0.001	< 0.001	< 0.001		

We had three light color treatments: cool white (peak 450 nm, range 410–740 nm), yellow (peak 587 nm, range 490–740 nm), and red (peak 617 nm, range 555–740 nm), as well as a control (the LED light switched off). Treatment colors were chosen based on the colors used in street lighting; cool white as in most modern LED lights, yellow light of older sodium lights, and red light that is used in streetlights that aim to be environment-friendly, especially to bats (Falchi et al. 2011; Spoelstra et al. 2017, Pagden et al. 2020). All of these had a relatively broad spectrum, similar to typical LED lighting, but unlike the narrow spectra of sodium lights (Elvidge et al. 2010). We used a spectrometer with a cosine corrector (FLAME-S, Ocean Insight, Orlando, USA) and the operating software OceanView (v. 1.6.7, Ocean Insight, Orlando, USA) to measure the intensity and spectrum of the artificial light at five locations within the arena (Table 1). We adjusted light intensity, using layers of color-foil, so that the intensity (photon flux) in the lit end of the arena (zones + 4 and + 5) was as similar between treatments as our adjustment methods allowed.

To observe male movement and orientation within the arena, we used a Raspberry Pi microcomputer connected to a compatible infrared camera module inserted into the roof of the arena, which transmitted the view of the arena to a screen outside of the arena. To make the arena visible in the dark control, it was illuminated, in all treatments, by five infrared LEDs (3 mm) attached to the roof of the arena. In order not to expose the focal males to artificial light before they were tested (e.g., by light from the monitoring screen or leaks when switching light sources between treatments), we only kept a few (5 or fewer) males inside the laboratory room at a time. They were kept in a cardboard box that shielded them from the sources of artificial light until they were swiftly transferred into the arena. The rest of the males were kept in a separate space, where they were exposed to natural light conditions. We ran the trials between 00:30 and 1:30, the reproductively active period of the local population. Notably, the natural levels of nighttime illumination in the study area during the glow-worm reproductive season are typically between 0.2 and 0.6 lx (3.2E + 11–9.60E + 11 photons/cm^2^/s), and in an open area up to 1 lx (1.7E + 12 photons/cm^2^/s) between 00:30 and 1:30, depending on weather conditions and the phase of the moon (Kivelä et al. 2023; personal observations, measured in 2024 in the study area using spectrometer and integrating sphere).

We tested one male at a time. We placed the male in the center of the arena on a round dish (⌀ 8 cm, edge 4 mm). We first waited for max 2 min for the focal male to move, then for max 2 min for it to leave the dish, and finally we tracked its movements in the arena for 2 min, noting the time it spent in each zone. Hence, for a male that completed the trial, the total time span from the initiation of the observation until it ended was 2–6 min. The males that left the dish within the allocated time were classified as active. The replicate was terminated if the focal male did not move within the first 2 min (classified as inactive), or if the male started to move but did not leave the dish within the next 2 min (in which case the male could not be classified as either active or inactive; N_control_ = 3, N_white_ = 1). We conducted a total of 50 white, 40 yellow, 39 red and 45 control replicates. The numbers of active and inactive males are reported in the results. We altered the order in which we ran the replicates, both within and between nights, so that after tracking an ‘active’ male, we always ran a replicate with one of the other light treatments.

### Statistical analyses

We analysed the data using R v. 4.2.0. (R Core Team 2022) and RStudio v. 2024.12.0 + 467 (Posit Team 2024) for macOS. To analyze the influence of light color on activity (the probability that males left the central dish), we used generalized linear mixed model (GLMM) with binomial error distribution (R package lme4 v. 1.1.29.; Bates et al. 2015), in which the response variable was whether the male left the dish, and the explanatory variable was the light condition (i.e., treatment: white/yellow/red/control). To account for variation among nights, we included date as a random effect.

To assess the effect of light conditions on male orientation after it had left the central dish, we calculated an orientation index based on the time and distance the male spent away from the central dish. In particular, we multiplied the time the male spent in each zone by the distance of that zone from the central dish (with the zone distance getting values from -5 to 5) and then divided the sum of the multiplied values with trial duration. The influence of light conditions on male orientation was analyzed using a linear mixed effects model (LMM) (R package nlme v. 3.1–157.; Pinheiro, J., Bates, D., R Core Team 2023) with the orientation index as a continuous response variable, the light condition as an explanatory variable, and date as a random effect. We verified model fit visually by checking the normal distribution of the residuals and the homoscedasticity of the residual variance. We excluded seven replicates (N_control_ = 1, N_yellow_ = 2, N_red_ = 4) in which the trial duration did not add up to 120 s, due to errors in recording the time. 

We did not record the exact time each trial was initiated. To account for a potential order effect, we calculated a starting time index for each trial (and hence male), based on the order and the total number of trials we ran each night. In particular, we multiplied the night-specific order number of each male’s trial by the maximum number of trials we tested in a night (16) and then divided it by the number of trials we ran that specific night (8–16). This procedure gave us a continuous variable with values between 1 and 16. We then included this index in the models as a fixed variable. However, the inclusion of this variable raised model AIC values without causing any qualitative changes to the results, and we thus dropped it from the final models.

## Results

### Activity (probability of moving to the arena)

The white light treatment had the lowest proportion of active males (males that started to move and then left the dish within the allocated time) (Table 2). The red light treatment had the highest proportion of active males (Tables 2, 3), with these two treatments being the only ones that significantly differed from each other (Tables 2, 3).

**Table 2:** Male glow-worm (*Lampyris noctiluca*) activity, i.e., inclination to leave the central dish, under different light conditions (treatments)

Treatment	Males (N)	Active (Left the center)	Inactive (Did not leave)	Active (%)
Control	42	35	7	83.3
White	49	35	14	71.4
Yellow	40	34	6	85.0
Red	39	35	4	89.7

**Table 3:** Pairwise comparisons of the probability that males left the central dish under different light conditions [white, yellow or red light or no light (control)]. Data were analyzed using a GLMM with binomial error distribution and date as random factor. N = 49 for white, 40 for yellow, 39 for red, and 42 for control

Pairwise comparisons of numbers of active males				
Treatment	Estimate	Std. Error	*Z*	*P*
White vs control	− 0.752	0.501	− 1.370	0.170
Yellow vs control	0.115	0.630	0.182	0.855
Red vs control	0.642	0.695	0.925	0.355
Yellow vs white	0.866	0.572	1.515	0.130
Red vs white	1.394	0.646	2.158	0.031
Red vs yellow	0.528	0.713	0.740	0.459

### Orientation in relation to the light

**Fig. 2:**
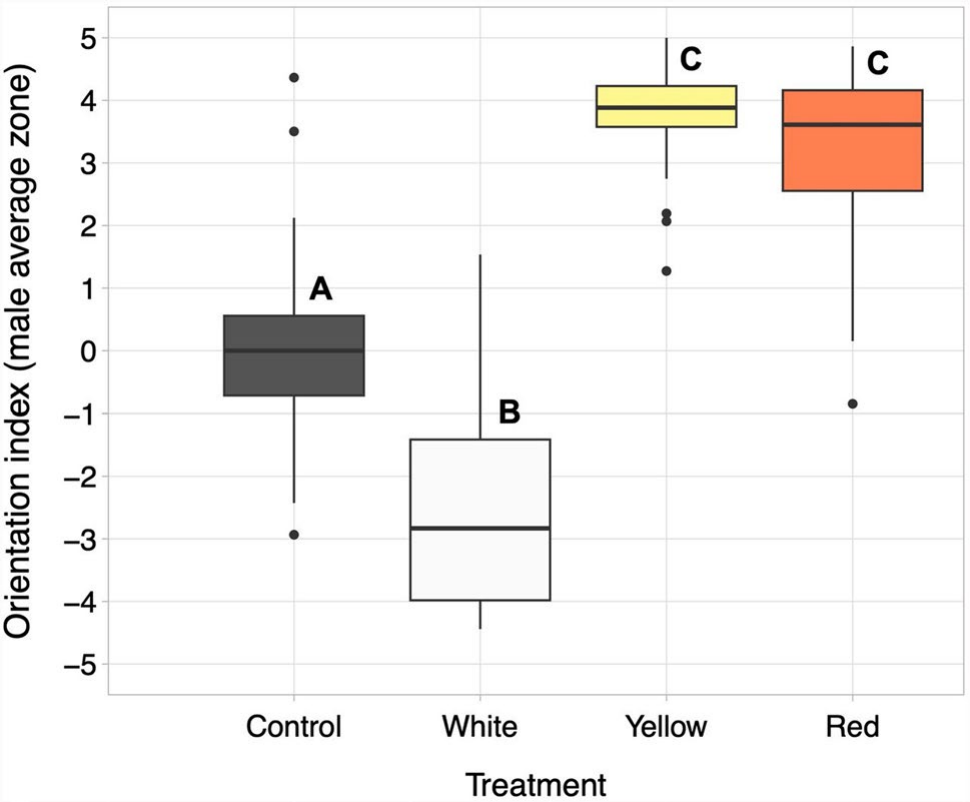
Male orientation in the experimental arena in relation to the color of light at one end of the arena, zones 4 and 5. Males started in zone 0, and their orientation index was calculated as the weighted average time spent in each zone after leaving the central dish. Treatments that do not share a letter (A–C) differ from each other (linear mixed effects model, α = 0.05). N = 34 for control, 35 for white, 31 for yellow, and 32 for red

Males in the yellow and red light treatments oriented their movements more toward the light source (i.e., spent on average more time in the lit end of the arena) than males in the white treatment and in the control (Fig. 2 and Table 4). Males in the white treatment oriented their movement more toward the dark end of the arena than males in the yellow and red treatments and in the control. Males in the control showed no preference for either end of the arena (Fig. 2). Males in the yellow and red light treatments did not differ from each other in their orientation (Fig. 2, Table 4).

**Table 4:** Pairwise comparisons of the orientation index (average zone location relative to arena center) of males that left the central dish under different light conditions [white, yellow or red light or no light (control)]. Data were analyzed using a LMM with date as a random factor. N = 35 for white, 32 for yellow, 31 for red, and 34 for control

Pairwise comparisons of male orientation				
Treatment	Estimate	Std. Error	*T*	*P*
White vs control	− 2.380	0.326	− 7.293	< 0.001
Yellow vs control	3.842	0.333	11.534	< 0.001
Red vs control	3.253	0.337	9.661	< 0.001
Yellow vs white	6.223	0.332	18.747	< 0.001
Red vs white	5.633	0.337	16.711	< 0.001
Red vs yellow	− 0.590	0.344	− 1.716	0.089

## Discussion

The results demonstrate that male glow-worms show strong positive phototaxis toward longer wavelength—yellow and red—light, and negative phototaxis toward shorter wavelength white light. In addition, males are less active under white light, especially when compared to red light. Hence, male glow-worms are responsive to a wide range of light colors, with their responses depending on the light spectrum.

The cause of the proportion of active males being the lowest in the white light treatment (but significantly so only compared to the red light treatment) may be that the light in that treatment was most similar to daylight and males are mostly inactive during daytime. Here, it is relevant to note that the proportion of active males being the highest in the red light treatment might have been influenced by light intensity being slightly higher in the red light treatment compared especially to the yellow light treatment, particularly in the central zones. We do not have repeated measurements of these small intensity differences to account for them statistically. In the dark control, males showed no preference for either section of the arena but instead often moved in circles, presumably because of the absence of light cues. The light conditions in the control treatment were darker than what mate-searching males are likely to experience in nature. We nevertheless chose this approach, because it allowed us to efficiently control for sources of orientation, other than light. Males did not perceive the infrared light used to observe them, judging by the fact that they did not appear to approach, avoid or orient themselves in relation to it.

Our finding that male glow-worms are attracted to red light aligns with previous anecdotal observations of males approaching a source of red light (Schwalb 1961). Similarly, male fireflies of the genus *Diaphanes* are attracted to red LEDs (Pacheco et al. 2016). The repulsion to white light combined with lower activity, in turn, aligns with previous findings of lampyrids being generally unresponsive to short-wavelength light (Buck 1937; Schwalb 1961; Lall & Worthy 2000; Booth et al. 2004). The spectral sensitivity of the common glow-worm is unknown, but males appear to possess photoreceptors that cover both short (< 500 nm) and long (> 500 nm) wavelengths (Booth et al. 2004). It is plausible that males can discriminate white light from the yellowish-green female glow (peak 550 nm, range 500–700 nm; De Cock 2004; Borshagovski et al. 2020), but that longer wavelengths, such as our yellow (peak 587 nm, range 490–740 nm) and red (peak 617 nm, range 555–740 nm) lights, resemble the glow of females to the degree that they trigger the same attraction response. In support of this conclusion, adding a red component to a green signal does not reduce its attractiveness to males (Booth et al. 2004). Thus, the attraction to longer wavelength light may form an evolutionary trap in environments lit by artificial light (Schlaepfer et al. 2002). It is likely to incur similar fitness costs as for moths attracted to short-wavelength light: exhaustion, injury, predation and missed mating opportunities (Frank 2006). Therefore, while male phototaxis toward the longer wavelengths of the yellowish-green female glow is adaptive under natural nighttime conditions, phototaxis toward these wavelengths in artificially lit environments is maladaptive. Given that in our experiment males did not initially see the actual light source but were nevertheless affected, it is likely that mere scattered light from shielded streetlamps or other similar light sources attracts male glow-worms.

These results indicate that artificial light of both short and long wavelengths can disrupt reproduction in the glow-worm and tuning broad-spectrum LED lights by filtering out either end of the spectrum does not provide an effective mitigation measure; females stop glowing and males fail to detect glowing females under shorter wavelengths (Ineichen & Rüttimann 2012; Elgert et al. 2020; Van den Broeck et al. 2021b; Kivelä et al. 2023), while males are attracted to longer wavelengths (this study). Hence, our findings contribute to the growing body of knowledge on the effects of light pollution on fireflies that can be utilized in the protection of this vulnerable and charismatic group of insects (Lewis et al. 2020; Owens & Lewis 2021). Nevertheless, more research is needed into the effects of narrowband or monochromatic light on glow-worms and other insects, especially as initial findings suggest that even narrow spectrum yellow and red light can disrupt firefly courtship signaling (Owens & Lewis 2021).

At a broader level, our results highlight the importance of considering multiple spectra and behaviors. While light with a certain wavelength peak may have no effect on a specific behavior, it may nevertheless influence other behaviors, and light with another peak wavelength could have yet a different effect on the behaviors. Hence, our findings underscore that differences among sexes (and life stages) need to be considered, especially when the sexes or life stages occupy different ecological niches and inhabit different environments, as is the case in many insects. Considering the large variation in spectral sensitivity among insects as well as other taxa, and that different behaviors within a species can vary in their spectral sensitivity (as shown in this study), spectral tuning should be employed with care. While warmer-toned light, with the blue part of the spectrum filtered out, may be less harmful for many species, other species may exhibit different or unexpected responses, such as the attraction of glow-worm males to long-wavelength light, shown in the current study. Documented effects of long-wavelength dominated light on other species include, for example, preferential attraction to amber light in click beetles and fungus flies (Deichmann et al. 2021), decreased foraging activity of Santa Rosa beach mice (*Peromyscus polionotus leucocephalus*) in the vicinity of yellow lights (Bird et al. 2004), and changes in the flight patterns of certain species of bats in response to even red streetlights (Zeale et al. 2018; Barré et al 2021). Thus, alternative mitigation measures to consider include reduced intensities and spatial and temporal distribution of ALAN, by shielding or dimming the light sources, using timers or motion sensors, or limiting the width of light spectra (Gaston et al. 2012; Longcore & Rich 2016; Owens et al. 2020). Limiting the amount of light will also save energy. Moreover, narrow spectrum or monochromatic light can in many situations be ecologically less harmful than broader spectrum light, when it reduces the number of species or behaviors sensitive to it (Gaston et al. 2012). The use of narrower spectra here could also have produced clearer answers about the effects of very specific light colors. However, outdoor lights, especially LEDs, are mostly broad-spectrum, which offers better color rendering and thus tends to elicit a higher sense of safety and comfort to humans than the narrower spectrum light from sodium lamps (Elvidge et al. 2010; Peña-García et al. 2015; Rea et al. 2017) although not necessarily higher visual acuity (Falchi et al. 2011; Lucas et al. 2014) . Here, it is relevant to note that LED technologies would also allow light outputs of narrow spectrum (Schubert & Kim 2005). 

To summarize, our results show that yellow and red artificial light attract male glow-worms, while white light repels them. The former may result in an evolutionary trap, if males are attracted to artificial light of longer wavelengths through the same mechanisms that allow them to efficiently locate the yellowish-green glow of females. While earlier research has indicated that species differ in their sensitivity to light of different wavelength spectra, our results show that behaviors within a species can also differ in sensitivity. While mate detection in glow-worms is most sensitive to artificial light dominated by shorter wavelengths, positive phototaxis is sensitive to ALAN of longer wavelengths. Thus, variation in spectral sensitivities, both among and within species as well as over different behaviors, needs to be considered when optimizing artificial outdoor lighting systems. Furthermore, we conclude that light color adjustments alone cannot solve the light pollution problem, and therefore, additional measures, such as reduction of artificial light’s spectrum width, intensity and spatial coverage, are also needed.

## Supplementary Information

Below is the link to the electronic supplementary material.

Supplementary file1 (DOCX 273 KB)

## Declarations

### Data availability

Data available from the Harvard Dataverse: https://doi.org/10.7910/DVN/0C0Y9B
