# Supplementary material for: Mitigating the light pollution problem via spectral adjustment: color-biased phototaxis in male glow-worms
Source: Oecologia. 2025 Jul 12;207(8):133. doi: 10.1007/s00442-025-05768-3 (PMC12255557; doi:10.1007/s00442-025-05768-3)
Supplement: Supplementary file 1 — Supplementary file1 (DOCX 273 KB) [file 442_2025_5768_MOESM1_ESM.docx]

**Mitigating the light pollution problem via spectral adjustment: color-biased phototaxis in male glow-worms**
Linnea Kivelä^a,b^, Christina Elgert^a,b^ , Topi K. Lehtonen^a,b^ and Ulrika Candolin^a,b^

^a^ Organismal and Evolutionary Biology, University of Helsinki, PO Box 65, 00014 Helsinki, Finland

^b^ Tvärminne Zoological Station, University of Helsinki, J.A. Palménin tie 260, 10900 Hanko, Finland

Correspondence:

Linnea Kivelä

linnea.kivela@helsinki.fi

Organismal and Evolutionary Biology, University of Helsinki, PO Box 65, 00014 Helsinki, Finland

**Supplementary material**

**Figure S1.** Spectral distributions for the light sources used in the white, yellow and red light treatments. Corresponding photon values (photons/cm^2^/s) were (1) white: 3.52E+12, (2) yellow: 3.74E+12, and (3) red: 3.83E+12.


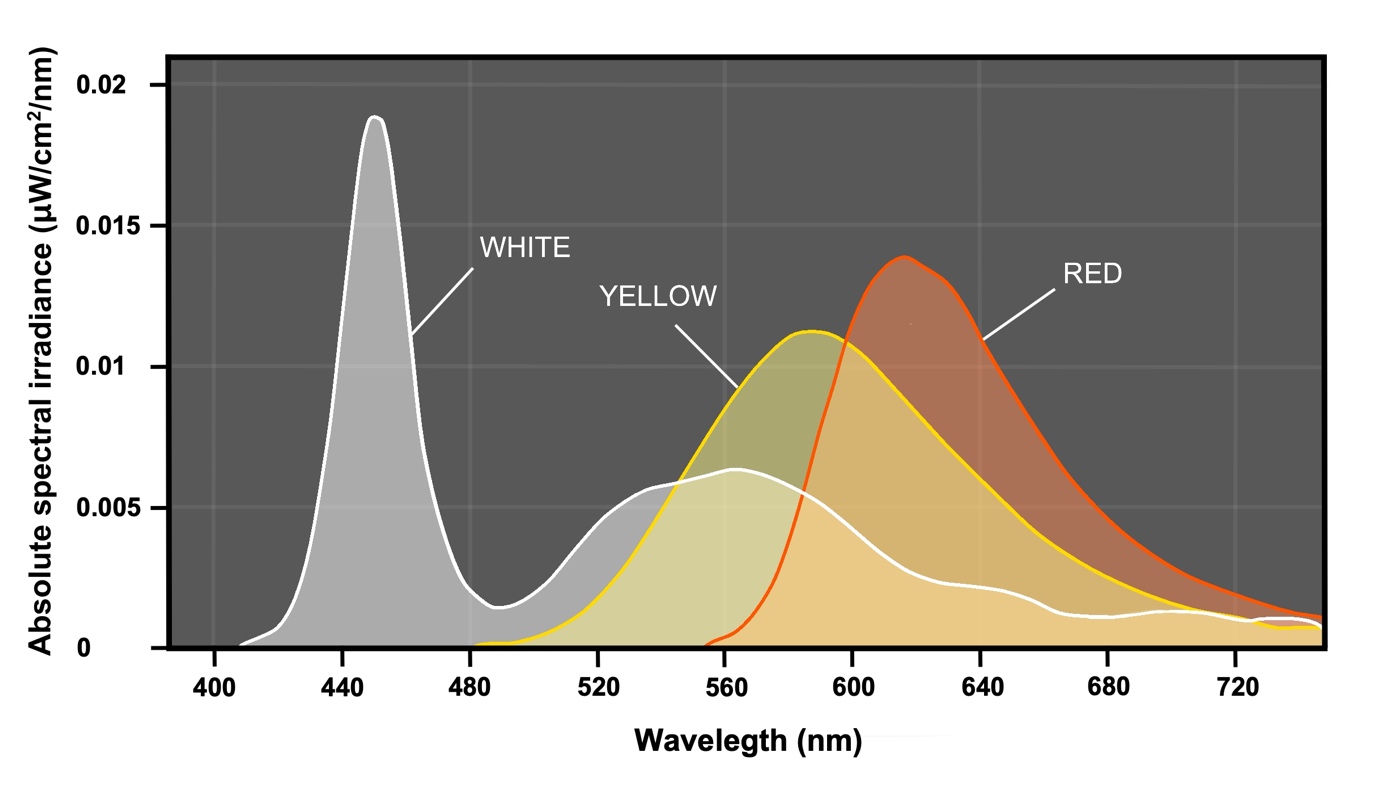


**Figure S2.** Photograph from inside of the experimental arena, taken from the dark end toward the lit end, in the yellow light treatment.

**
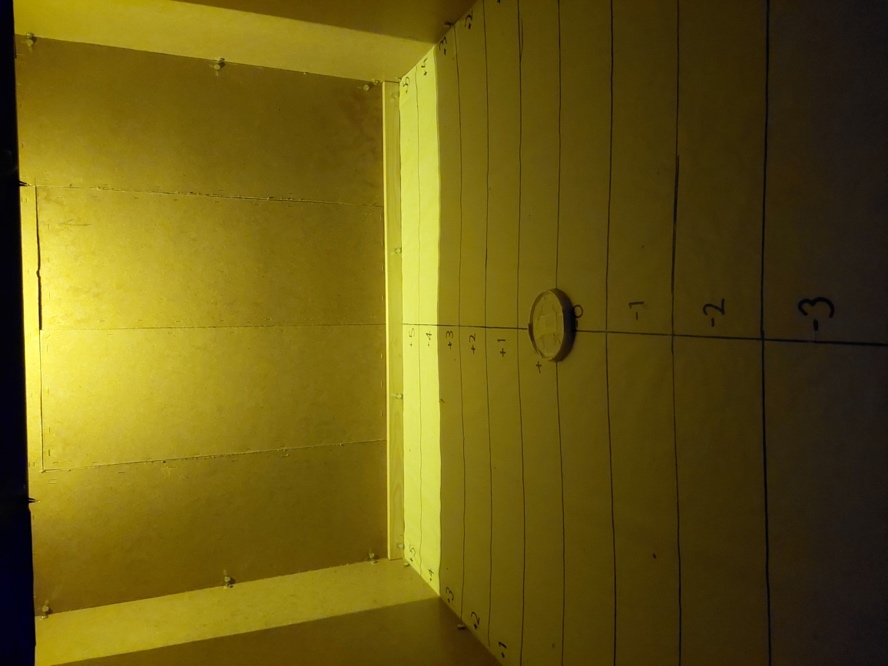
**
